# Low-Temperature Sintering of Ag Composite Pastes with Different Metal Organic Decomposition Additions

**DOI:** 10.3390/ma16062340

**Published:** 2023-03-14

**Authors:** Zixuan Xu, Xun Liu, Junjie Li, Rong Sun, Li Liu

**Affiliations:** 1 School of Materials Science and Engineering, Wuhan University of Technology, Wuhan 430070, China; 303909@whut.edu.cn; 2 Shenzhen Institute of Advanced Technology, Chinese Academy of Sciences, Shenzhen 518100, China; xun.liu@ac-semi.com (X.L.); rong.sun@siat.ac.cn (R.S.); 3 The Institute of Technological Science, Wuhan University, Wuhan 430072, China; 4 State Key Laboratory of Advanced Welding and Joining, Harbin Institute of Technology, Harbin 150001, China

**Keywords:** MOD, Ag composite pastes, low-temperature sintering, shear strength

## Abstract

Rapid developments in wide-bandgap semiconductors have led to the demand for interconnection materials that can withstand harsh conditions. In this study, novel Ag composite pastes were developed with the assistance of metal organic decomposition (MOD) to significantly reduce the sintering temperature of commercial Ag pastes. The effects of the decomposition characteristics of different MODs on the microstructure, morphology, and the shear strength of the Ag-sintered joints were systematically investigated. Additionally, the low-temperature sintering mechanisms of the MOD-assisted Ag composite pastes were studied and proposed. Among all the MODs studied, the one consisting of propylamine complexed with silver oxalate demonstrated the best performance due to its ability to form Ag nanoclusters with the smallest size (~25 nm) and highest purity (~99.07 wt.%). Notably, the bonding temperature of the MOD-modified Ag pastes decreased from 250 °C to 175 °C, while the shear strength increased from 20 MPa to 40.6 MPa when compared to the commercial Ag pastes.

## 1. Introduction

The exponential growth of electronic devices has resulted in their pervasive use [1,2,3]. Recent advances in power electronics, including spacecraft, renewable energy vehicles, and high-speed trains, have propelled the demand for electronic packaging towards smaller, faster, and more efficient designs that are suitable for use in harsh conditions. The applications of wide-bandgap (WBG) semiconductors, such as SiC and GaN, continue to expand due to their advanced properties in severe conditions, typically involving temperatures of around 250 °C [4,5]. Consequently, there is a need for die-attach materials that can endure long periods of exposure to temperatures close to or even exceeding 250 °C. However, conventional Sn-based solders are inadequate for application at high temperature. Therefore, it is of paramount importance to develop novel die-attach materials that can facilitate low-temperature joining and also withstand high-temperature working environments [6,7,8].

Ag nanoparticles are widely used as lead-free die attachments due to their relatively high conductivity, superior fatigue properties, and high melting point [9,10,11]. However, the high sintering temperature (~250 °C) and long sintering time (30~60 min) associated with their production limit their widespread application [12]. Moreover, the high temperature and long sintering time causes thermal stress inside the joint, which may affect its reliability. Therefore, reducing the sintering temperature of silver paste is crucial to enable the large-scale application of this technology. Numerous attempts have been made to lower the sintering temperature by decreasing the size of the Ag particles, but this approach often leads to the agglomeration of Ag nanoparticles at room temperature during synthesis and storage periods. Moreover, synthesizing Ag nanoparticles with diameters below 20 nm is challenging [13,14]. Recent studies have suggested that adding silver salts, such as AgO and AgC_2_O_4_, to Ag pastes can further decrease the sintering temperature by forming Ag nanoparticles through decomposition. The method of achieving low-temperature sintering by adding Ag_2_O particles provides a new research direction: developing an addition that can decompose and generate smaller nano-Ag particles in situ at low temperatures [15,16].

The field of smart materials is rapidly developing, with some materials exhibiting the ability to produce nanoparticles at low temperatures [17]. Ag-based MOD (metal organic decomposition) is one such flexible electronic material that produces Ag nanoparticles upon exposure to light or heat (100–200 °C) [18,19,20,21,22]. MOD ink is typically prepared using silver salts (as precursors), organic amines (as complexing agents), and solvents and reducing agents, such as alcohols and organic acids. However, MOD ink often contains large amounts of organic solvents. To address this, the main component (MODs) can be extracted from MOD ink and added to Ag pastes. The MODs can be decomposed on the surface of large-sized Ag particles at low temperatures, generating smaller Ag clusters. These clusters can fill the gaps between large-sized Ag particles and promote low-temperature bonding areas of Ag pastes. It is worth nothing that different MOD inks exhibit different properties due to the varying precursors or organic amines used in their preparation, resulting in different properties of the resulting silver–ammonia complex (MODs). At present, little research has been conducted on the properties of different MODs, and their potential as additions for achieving low-temperature sintering of silver paste warrants further investigation.

This study aims to provide a comprehensive understanding of the low-temperature sintering behavior of silver pastes with various MOD additions. To this end, seven types of MODs were synthesized and characterized to investigate their decomposition characteristics. Furthermore, the microstructure, morphology, and shear strength of Ag-sintered joints modified by these MODs were thoroughly examined. Finally, a low-temperature sintering mechanism of MOD-assisted Ag composite pastes was studied and proposed.

## 2. Materials and Methods

### 2.1. Preparations of MODs

In this study, a range of MODs with varying amines were synthesized to assist sintering at a low bonding temperature. Specifically, MOD1–MOD7 are silver–ammine complexes formed through complexation reactions of the same precursor with different organic amines. The precursor employed for all complexes was silver oxide, while the organic amines used included ethylamine, propylamine, butylamine, hexylamine, octylamine, ethylenediamine, and 1, 2-propylenediamine. The synthesis conditions were kept identical for all complexes. Silver oxalate was used as the precursor due to its ability to decompose into solid silver and carbon dioxide. Different monoamines and diamines were employed to investigate their impact on the properties of MODs and Ag-sintered joints. The reactions involved in MOD synthesis can be represented by Equations (1) and (2).

Monoamines: Ag_2_C_2_O_4_ + 4C_n_H_2n+3_N → [Ag(C_n_H_2n+3_N)_2_]_2_C_2_O_4_ (1)

Diamines: Ag_2_C_2_O_4_ + 2C_n_H_2n+4_N_2_ → (AgC_n_H_2n+4_N_2_)_2_C_2_O_4_ (2)

**Figure 1:**
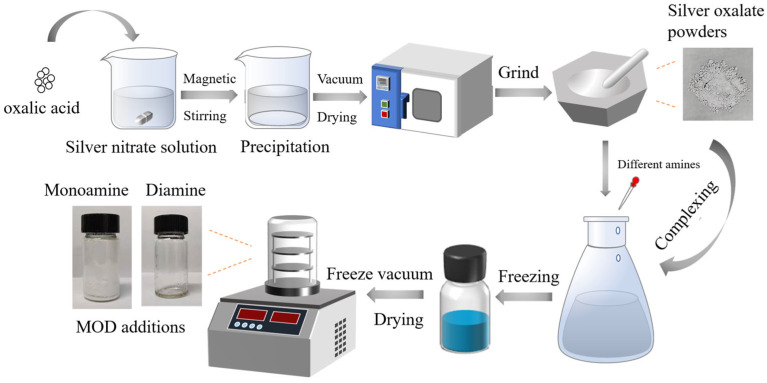
Schematic illustration of MOD preparations.

To obtain the silver oxalate powders, a solution of oxalic acid (0.5 mol/L) was gradually added to a solution of silver nitrate (0.8 mol/L) to generate silver oxalate precipitations. The precipitates were collected through centrifugation at 5000 rpm for 3 min and subsequently dried in a vacuum oven at 40 °C for 6 h. Meanwhile, different organic amines, including ethylamine, propylamine, butylamine, hexylamine, octylamine, ethylenediamine, and 1, 2-propanediamine, were added in the tert-butanol–water co-solvent, which was used as an ink solvent. To avoid the formation of unwanted byproducts due to the exothermic reaction between silver oxalate and the various amine compounds at room temperature, a low-temperature electromagnetic stirring approach at 5 °C was adopted to obtain the different MOD inks. The resulting MOD additions were obtained through freeze-drying of the MOD inks. The schematic of the preparation of the MOD additions is illustrated in Figure 1.

### 2.2. Fabrication of Ag Composite Pastes and Sintering Joints

**Figure 2:**
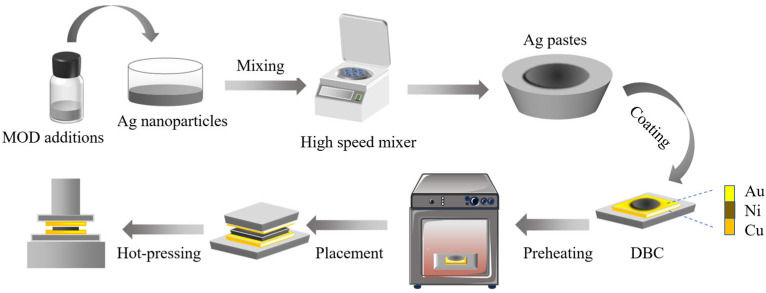
Preparation process of Ag pastes and sintered joints.

The Ag composite pastes were prepared by combining 0.2 g of commercial Ag powder (average diameter: 60 nm), 0.05 g of self-prepared MODs, and 0.05 g of organic solvent. The organic solvent was formulated by mixing isopropanol, ethylene glycol, and terpineol in a mass ratio of 2:2:1. Subsequently, the Ag composite pastes were stencil printed evenly onto the DBC (direct bonding copper) surface in two different sizes: 6 mm × 6 mm and 3 mm × 3 mm. Finally, the Ag joints with a sandwich structure were sintered at 175 °C for 10 min under 10 MPa of pressure. The entire preparation process of Ag pastes and sintered joints is presented in Figure 2.

### 2.3. Characterization and Measurement

A UV–vis spectrophotometer (Shimadzu UV-1800, Shanghai, China) was used to obtain the ultraviolet–visible (UV–vis) absorption spectra of the MODs. The coordination and complexation of the amine groups in the MODs were analyzed using Fourier transform infrared (FT-IR) spectra (IRPrestige-21, Kyoto, Japan). Moreover, a simultaneous thermogravimetric analyzer (TG-DSC, STA449 F3, Jupiter, Selbu, Germany) was employed to analyze the thermal behaviors of the MODs, silver oxalate, and Ag pastes. The DSC tests were carried out by heating the samples from 30 °C to 300 °C with a heating rate of 10 °C/min in an air atmosphere. Subsequently, the shear strength of Ag-sintered joints was evaluated by a DAGE 4000 (Westlake, OH, USA). The ASTM (American Society for Testing and Materials) standard used for the shear test was ASTM B831, with sample dimensions of 3 × 3 mm^2^ and 6 × 6 mm^2^. Furthermore, a scanning electron microscope (SEM, FEI Nova Nana SEM 450, Portland, OR, USA) was applied to observe the morphological features of the Ag-sintered joints from their fractured surface. The surface morphology of the Ag-sintered films was observed using SEM, and the porosity of the sintered films was measured by Image J software. Additionally, the morphology and element analysis of the MODs were also carried out by the SEM equipped with energy-dispersive spectroscopy. The sheet resistance of the sintered silver film was evaluated using the four-point probe method.

## 3. Properties of MODs

**Figure 3:**
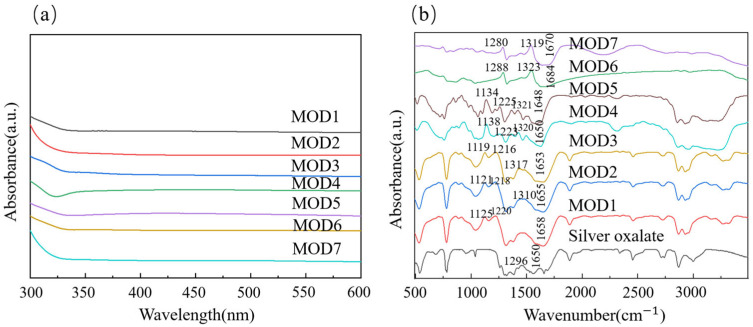
UV–visible and IR spectra of different MODs: (**a**) UV–visible spectra of different MODs, (**b**) IR spectra of different MODs.

UV–visible and IR spectroscopic techniques were used to investigate the chemical compositions of various MODs in the initial state. Notably, the absence of absorption peaks for Ag nanoparticles in the wavelength range of 380–450 nm in Figure 3a suggested the formation of a complex [23]. Furthermore, the FT-IR results depicted in Figure 3b revealed that each of the MOD1–MOD7 samples exhibited infrared absorption peaks of the amino group (-NH_2_) in the range of 1110–1300 cm^−1^, indicating that the amino groups had formed coordination bonds with Ag ions. In comparison to the C-based peaks of silver oxalate, which were observed at 1296 cm^−1^ and 1650 cm^−1^, the C-based peaks of MOD1-MOD7 were found to be slightly right-shifted to 1313–1325 cm^−1^ and 1655–1685 cm^−1^, respectively. This shift can be attributed to the lone pair of electrons on the amino group entering the empty orbital on the silver atoms, resulting in an increase in the electron cloud density and a shift to a higher wave number. Therefore, it can be concluded that the silver oxalate and all amines were completely mixed to obtain MODs.

**Figure 4:**
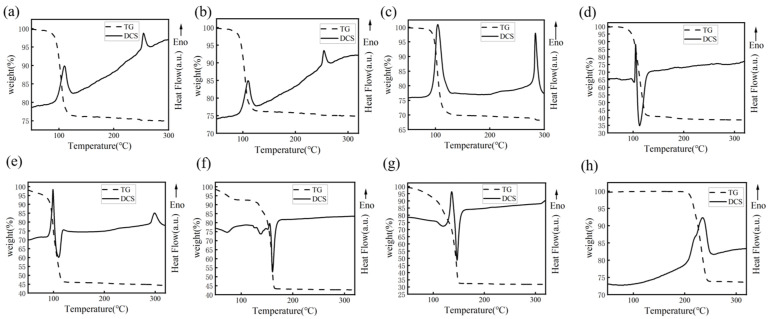
TG-DSC curves of different MOD and silver oxalate (Arrow direction represents endothermic reaction): (**a**) MOD1, (**b**) MOD2, (**c**) MOD3, (**d**) MOD4, (**e**) MOD5, (**f**) MOD6, (**g**) MOD7, (**h**) silver oxalate.

The thermal behaviors of seven MODs containing different amines were studied using simultaneous thermal analysis. It can be seen in Figure 4h that the endothermic peak of silver oxalate (~240 °C) was significantly higher than the peaks exhibited by the MODs [20], which had endothermic peaks below 150 °C. Furthermore, a sharp loss in mass was observed during these peak temperatures, suggesting that the MODs have low decomposition temperatures. In Figure 4a–e, the MODs containing monoamines exhibited endothermic peaks in the temperature range of 95–125 °C. Among these, MOD4 and MOD5 had decomposition temperatures of approximately 102 °C and 95 °C, respectively, which were slightly lower than those of MOD1–MOD3 (112 °C, 110 °C, and 106 °C). As for the MODs with diamine, they exhibited similar thermal behaviors, but with higher decomposition temperatures of about 150 °C and 145 °C, as shown in Figure 4f,g, respectively. Therefore, the MODs can be decomposed at low temperature (<200 °C) and have the potential for low-temperature sintering.

Studies have shown that the decomposition temperature of silver–amine complexes can be influenced by the type of amine and its alkyl chain length [24]. Compared to monoamine complexes, diamine complexes, such as MOD6 and MOD7, have a higher thermal stability and decomposition temperature due to their electron-giving ability. In cases where amines have the same number of amino groups, the thermal stabilities of silver–amine complexes are primarily determined by steric hindrance, which decreases as the size of the side groups in the amines increases. Thus, when amines with long carbon-chain lengths, but the same number of amino groups, such as octylamine, were used to prepare the ink, the corresponding ink exhibited a significant decrease in its decomposition temperature.

**Figure 5:**
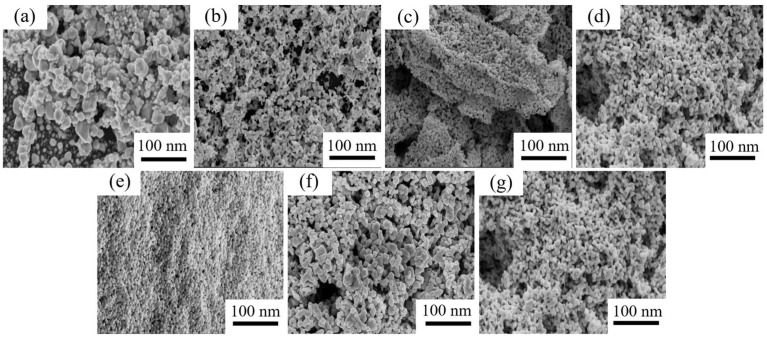
SEM images of sintered products of different MOD: (**a**) MOD1, (**b**) MOD2, (**c**) MOD3, (**d**) MOD4, (**e**) MOD5, (**f**) MOD6, (**g**) MOD7.

**Figure 6:**
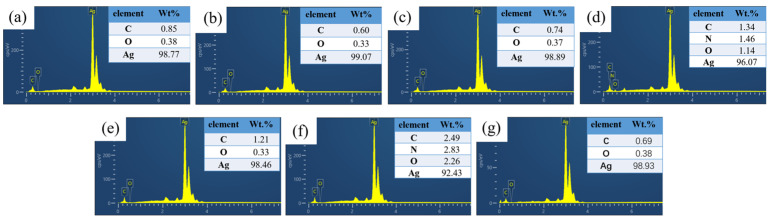
EDS results sintered products of different MOD: (**a**) MOD1, (**b**) MOD2, (**c**) MOD3, (**d**) MOD4, (**e**) MOD5, (**f**) MOD6, (**g**) MOD7.

The morphology and chemical composition of MOD films were characterized using SEM and EDS analysis, as shown in Figure 5 and Figure 6. The sintered products of all MODs were found to be Ag clusters with different sizes and morphologies. Moreover, the Ag content of the sintered products varied from the EDS analysis results. For instance, the MOD1 and MOD6 sintered products were observed to be Ag clusters with average sizes of 67.38 nm and 70.75 nm, respectively, with relatively low Ag contents of 98.89 wt.% and 96.91 wt.%, respectively. As shown in Figure 5b,c,g and Figure 6b,c,g, the particle sizes of the MOD2, MOD3, and MOD7 samples decreased significantly, with average sizes of 24.89 nm, 29.81 nm, and 25.6 nm, respectively. Moreover, their Ag contents were the highest, at 99.07 wt.%, 98.77 wt.%, and 98.93 wt.%, respectively. Conversely, the particle sizes of the sintered products of MOD4 and MOD5 increased slightly, with average particle sizes of 35.16 nm and 37.3 nm, respectively. However, the Ag contents of MOD4 and MOD5 were the lowest, with mass rations of 92.43 wt.% and 90.07 wt.%, respectively. Additionally, EDS results indicated the presence of an N element in both MOD4 and MOD5, which suggested that hexylamine and octylamine, with higher boiling points, were not completely volatilized [25].

The size and size distribution of Ag clusters have a significant impact on the required sintering temperature. Smaller particles require a lower sintering temperature. Moreover, a narrow size distribution promotes faster sintering at a lower temperature, as it allows for more even particle accumulation and larger contact areas between particles, promoting diffusion and coalescence. In contrast, a wide size distribution leads to uneven accumulation and decreased contact areas between particles, hindering diffusion and coalescence, and thus requiring a higher sintering temperature and a longer sintering time to achieve the desired bonding and densification.

The morphology of the Ag clusters that form during the sintering process varies among different MOD systems. The formation mechanism of these Ag clusters is closely related to the decomposition behaviors of the MODs. The thermal decomposition of the MODs can be described by the reaction formulas presented in Equations (3) and (4).

Monoamines: [Ag(C_n_H_2n+3_N)_2_]_2_C_2_O_4_→Ag(s) + C_n_H_2n+3_N(g) + CO_2_(g) (3)

Diamines: (AgC_n_H_2n+4_N_2_)_2_C_2_O_4_→Ag(s) + C_n_H_2n+4_N_2_(g) + CO_2_(g) (4)

**Figure 7:**
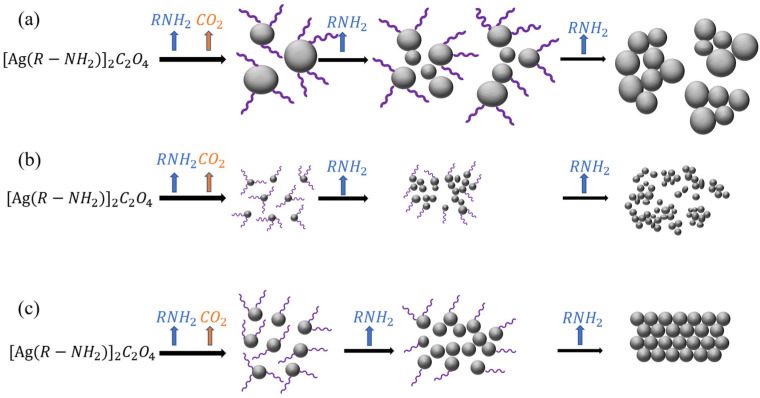
Decomposition mechanisms of different MODs: (**a**) MOD1, MOD6; (**b**) MOD2, MOD3, MOD7; (**c**) MOD4, MOD5.

Based on the analysis of the sintered products of different MODs in Figure 7, three distinct decomposition mechanisms can be identified. The first mechanism, exemplified by MOD1 and MOD6 (Figure 7a), involves high decomposition temperatures above the boiling points of corresponding ethylamine and ethylenediamine. In this case, the amines quickly evaporate, leaving the Ag nanoparticles unprotected and promoting the nucleation and growth of large Ag particles. As a result, isolated Ag islands are formed due to volume contraction, as also observed in previous work with MOD-synthesized silver tartrate [26]. This decomposition mechanism results in relatively fewer organic residues. In the second mechanism (MOD2, MOD3, and MOD7; Figure 7b), the decomposition temperatures are close to the boiling points of propylamine, butylamine, and 1, 2-diaminopropane, respectively. Here, small Ag nanoparticles of 20–30 nm diameter are formed with protection from the amine, making it more difficult for these small Ag particles to aggregate and form large-sized particles [27]. Consequently, fewer large particles are formed, resulting in the lowest number of organic residues. The final mechanism (MOD4 and MOD5; Figure 7c) is characterized by the high boiling points of hexylamine and octylamine, respectively. In this case, the MODs decompose prior to the evaporation of the amines, leaving a large amount of amine residual in the MOD matrix. The presence of those long chain-like structures of amine residual compresses the Ag nanoparticles, preventing further growth and leading to the formation of a dense microstructure, as observed in Figure 5d,e. The different decomposition mechanisms result in the formation of spherical nanoclusters with varying sizes and densities. The size of these nanoclusters affects grain growth, as smaller nanoclusters tend to form more grain boundaries, while larger nanoclusters tend to form fewer grain boundaries. A higher number of grain boundaries allows for more diffusion between grains, requiring less energy and reducing the sintering temperature. The density of the spherical nanoclusters also affects the sintering process. Higher densities improve the interaction and bonding between nano silver clusters, promoting the sintering process. However, high density can also lead to excessive sintering and agglomeration, which is not conducive to sintering.

## 4. Performance and Assisted Sintering Mechanism of Ag Composite Pastes

**Figure 8:**
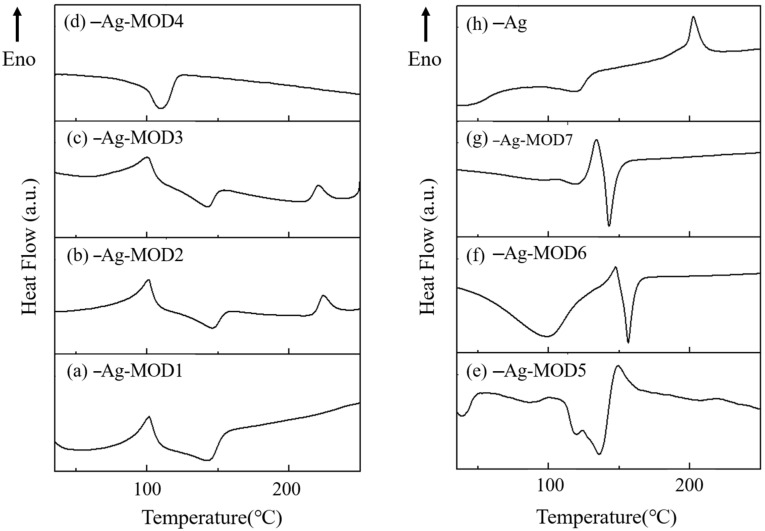
DSC curves of different Ag composite pastes (Arrow direction represents endothermic reaction): (**a**) Ag/MOD1, (**b**) Ag/MOD2, (**c**) Ag/MOD3, (**d**) Ag/MOD4, (**e**) Ag/MOD5, (**f**) Ag/MOD6, (**g**) Ag/MOD7, (**h**) Ag.

The Ag pastes containing various MOD additions (designated as M1–M7) and pure Ag pastes were sintered in a dry air environment to investigate the sintering effects of MODs, the results were shown in Figure 8. The additions of MODs resulted in the appearance of distinct endothermic peaks below 150 °C, which were absent in pure Ag pastes. Conversely, the endothermic peak of pure Ag pastes was observed at a higher temperature of over 200 °C. It is worth noting that most pure Ag pastes require sintering temperatures above 200 °C, typically around 250 °C [28]. Thus, the addition of MODs successfully reduced the endothermic peaks and enhanced thermal properties, demonstrating their potential in low-temperature sintering applications. Overall, the comparative analysis confirms that the MOD additions in the Ag composite paste exhibit the ability to decompose at lower temperatures.

**Figure 9:**
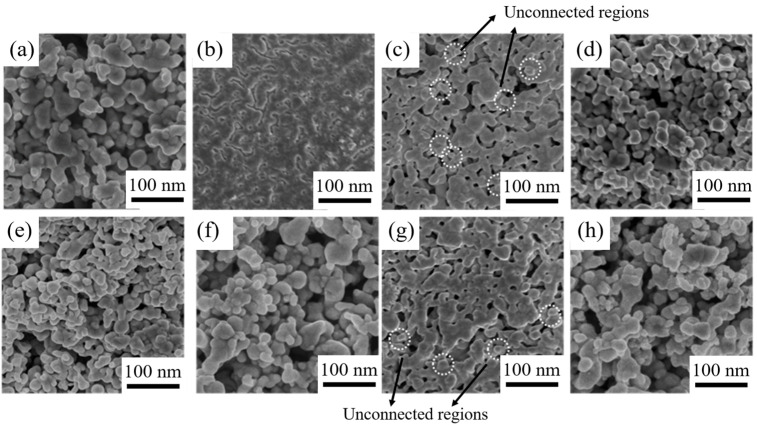
Morphology of Ag-sintered films of different composite pastes with: (**a**) MOD1, (**b**) MOD2, (**c**) MOD3, (**d**) MOD4, (**e**) MOD5, (**f**) MOD6, (**g**) MOD7, (**h**) pure Ag powders.

The sintered Ag films obtained from M1–M7 and pure Ag pastes at 175 °C are shown in Figure 9. As discussed earlier, Ag composite pastes containing MOD2, MOD3, and MOD7 exhibit the highest amount of neck joining points. Among these samples, the decomposition products of MOD2 possess the highest Ag content and the smallest particle size. The M2 sintered film displays the best interconnection phenomenon, with no distinct boundary between large Ag particles and a film porosity of 5.13%. Necking formation can also be observed in the M3 and M7 sintered films, but some unconnected regions indicated by the marked dashed circles are still present. The film porosities for M3 and M7 are 14.83% and 12.76%, respectively. However, the sintered structure of the remaining samples, including M1, M4, M5, M6, and pure Ag pastes, are notably poor, with film porosities of 52.34%, 45.28%, 47.36%, 55.63%, and 58.72%, respectively. The incomplete sintering of numerous Ag nanoparticles in these samples can be attributed to their relatively large particle size and impurity residues.

**Figure 10:**
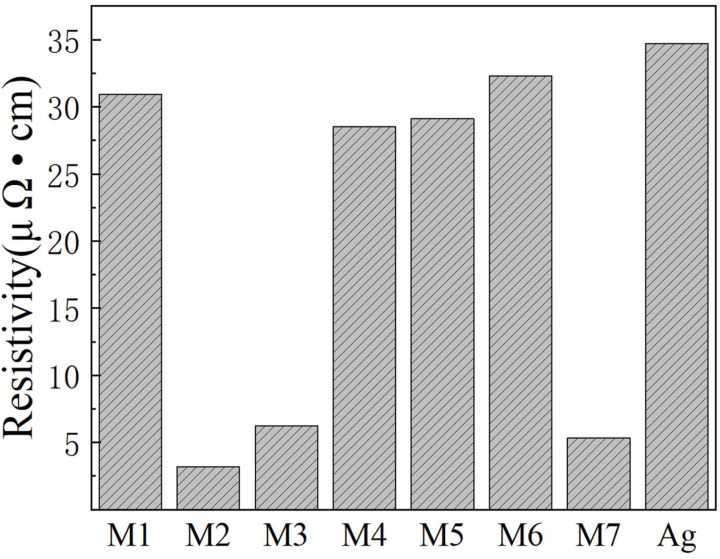
Resistivity of different Ag paste-sintered films.

Figure 10 illustrates the resistivity of the sintered silver film, which is closely related to its microstructure. Among the tested samples, the M2, M3, and M7 sintered silver films exhibit resistivities of 3.17, 6.24, and 5.33 μΩ·cm, respectively. Notably, the M2 sintered silver film demonstrates the smallest resistivity and a dense sintered structure. In contrast, the resistivities of M4 and M5 films are 28.52 μΩ·cm and 29.10 μΩ·cm, respectively. These results suggest that the resistivity of the film increases with porosity, and the impurity of electrons in the film also affects the current transmission. The relatively larger porosity of M1 and M6 sintered films results in resistivities (30.93 and 32.31 μΩ·cm, respectively) that are similar to that of the sintered conjunctival film without a silver paste sintering agent (34.68 μΩ·cm). These observations imply that the resistivity of the sintered film is mainly influenced by the porosity of the sintered film and the impurity content of MOD decomposition products. Specifically, large holes and high impurity content can hinder current transmission in the sintered film.

**Figure 11:**
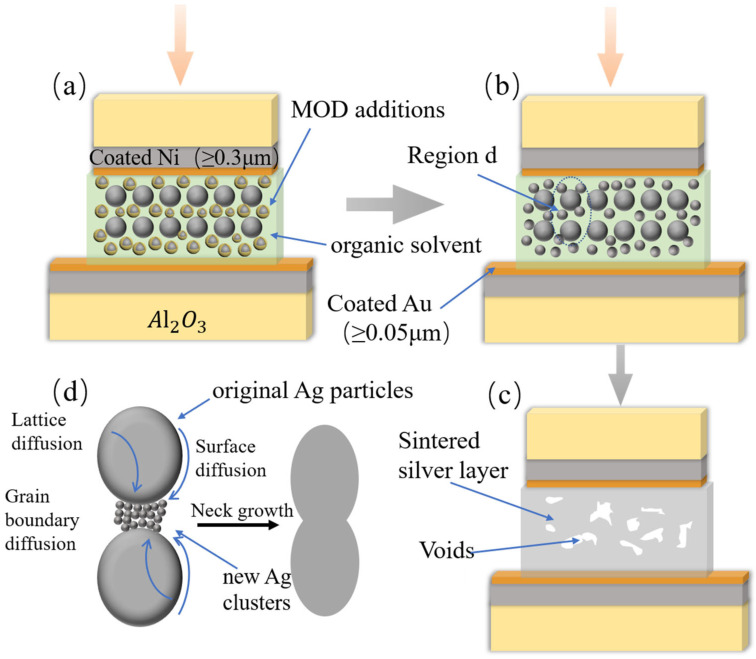
(**a**–**c**) The schematic diagram of a MOD-assisted sintering mechanism ((**d**) presents an enlarged view of a specific area in (**b**)).

Drawing upon the results and analyses presented above, a low-temperature sintering mechanism for the MODs can be proposed, as illustrated in Figure 11. During the sintering process, the thermal decomposition of the MOD additions generates Ag clusters in the nanoscale, which tend to aggregate around the original Ag particles to initiate grain boundary diffusion and joining. Since grain boundary diffusion requires lower activation energy in comparison with lattice and surface diffusion [29], the appearance of a higher degree of grain boundary diffusion substantially reduces the sintering temperature. Additionally, the distance between the Ag particles is significantly reduced, therefore promoting the diffusion and joining of these particles. Ultimately, this leads to the formation of a relatively dense sintered structure, as observed in Figure 9b.

## 5. Mechanical Properties of Ag-Sintered Joints

**Figure 12:**
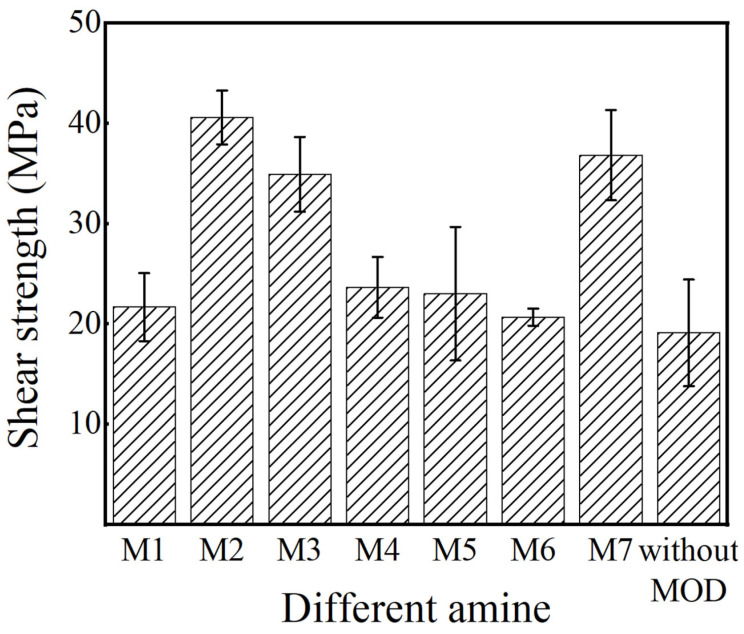
Shear strength of different Ag sintering joints: M1–M7 and pure Ag without MOD.

The mechanical properties of the Ag-sintered joints were analyzed with respect to the effects of the MODs. As shown in Figure 12, the addition of MODs resulted in Ag-sintered joints with higher shear strength than pure Ag-sintered joints. The shear strengths of the M1, M4, M5, and M6 samples, which have poor sintered morphology, large Ag size, and low Ag content, were only slightly increased to approximately 23.66 MPa, 23.02 MPa, 22.88 MPa, and 20.69 MPa, respectively, compared to the shear strength of pure Ag-sintered joints (~19.11 MPa). Conversely, due to their small Ag particle size and high Ag content, the shear strengths of the M2, M3, and M7 samples increased to 40.6 MPa, 34.94 MPa, and 36.84 MPa, respectively. Among all the samples, the Ag-sintered joints with MOD2 exhibited the highest shear strength due to the significant grain boundary diffusions.

**Figure 13:**
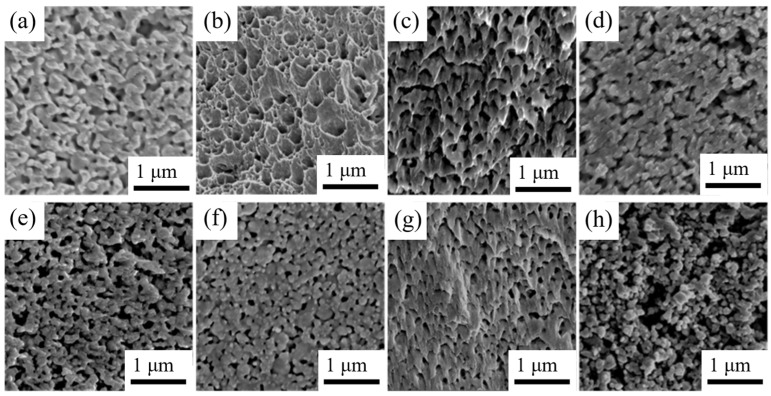
The fracture structures of different Ag sintering joints: (**a**) Ag/MOD1, (**b**) Ag/MOD2, (**c**) Ag/MOD3, (**d**) Ag/MOD4, (**e**) Ag/MOD5, (**f**) Ag/MOD6, (**g**) Ag/MOD7, (**h**) Ag.

Figure 13 displays the fracture surfaces of the aforementioned Ag-sintered joints after shear testing. Two distinct fracture characteristics are observed at the fractured surface of these sintered joints. For the sintered joints with higher shear strength, large areas of plastic deformation structure are visible in Figure 13b,c,g. The plastic deformation typically occurs in Ag-sintered joints with low porosity [30], leading to the destruction of numerous ligaments and grains. In the remaining samples with lower shear strength, their fracture morphologies (M1, M4–6) in Figure 13a,d–f exhibit no apparent plastic deformation, but rather hole-dominated intergranular fractures and phase interface fractures, similar to the pure Ag-sintered joints without MOD additions. The size and depth of dimples on the fracture surfaces of sintered joints were observed to vary significantly. This variation can be attributed to the sintering behavior of the silver particles during the process. The silver particles are heated to a particular temperature during sintering, leading to their softening and fusion. As the temperature increases further, the particles undergo deformation and necking, leading to elongation and merging. The size and depth of the dimples are linked to the degree of necking that occurs during sintering. Narrower necks between particles lead to shallower and smaller dimples, while wider necks result in deeper and larger dimples. The shape and size of the necks depend on various factors, such as the shape and size of the silver particles, as well as the sintering temperature and time. Furthermore, the presence of impurities or defects in the silver particles can affect the sintering behavior and dimple formation. These impurities and defects can cause uneven heating, resulting in uneven deformation and necking, leading to an irregular distribution of dimples on the fracture surface.

## 6. Conclusions

In this study, we aimed to develop a new chip interconnection material that meets the requirements of low-temperature interconnection, high working temperature, and high mechanical strength of power devices. To achieve this goal, we proposed a novel MOD addition that can decompose at low temperature to form nano-sized Ag particles, therefore significantly reducing the sintering temperature from 250 °C to 175 °C and increasing the shear strength from 19.11 MPa to 40.6 MPa compared to commercial pure Ag pastes without MODs. The effectiveness of the MOD addition was attributed to the close match between the decomposition temperature of MOD2 and the boiling point of propylamine, which protects the nano-Ag particles from aggregation and growth into larger sizes, resulting in the Ag clusters with the highest content (~99.07 wt.%) and smallest particle size (~25 nm) of all the additions tested. These new Ag clusters were found to gather around the original Ag particles to initiate grain boundary diffusion and joining, leading to the formation of a coarse necking joining structure with high bonding areas at low temperatures. The use of MOD additions in composite silver paste resulted in a higher-strength joint at 175 °C, making it a promising material for low-temperature interconnection of power devices. Overall, our findings demonstrate the potential of MODs as an effective means to enhance the mechanical properties of interconnections in power devices.

## Data Availability

The data that support the findings in this study are available from the corresponding author upon reasonable request.
